# Congenital abdominal wall defects and cryptorchidism: a population-based study

**DOI:** 10.1007/s00383-021-04863-9

**Published:** 2021-01-31

**Authors:** Arimatias Raitio, Johanna Syvänen, Asta Tauriainen, Anna Hyvärinen, Ulla Sankilampi, Mika Gissler, Ilkka Helenius

**Affiliations:** 1 Department of Paediatric Surgery, University of Turku and Turku University Hospital, Kiinamyllynkatu 4-8, 20521 Turku, Finland; 2 Department of Paediatric Surgery, Kuopio University Hospital, Puijonlaaksontie 2, 70210 Kuopio, Finland; 3 Department of Paediatric Surgery, University of Tampere and Tampere University Hospital, Elämänaukio, Kuntokatu 2, 33520 Tampere, Finland; 4 Department of Paediatrics, Kuopio University Hospital, Puijonlaaksontie 2, 70210 Kuopio, Finland; 5 Information Services Department, Finnish Institute for Health and Welfare, Mannerheimintie 166, PL 30, 00271 Helsinki, Finland; 6 Department of Neurobiology, Care Sciences and Society, Karolinska Institute, Solnavägen 1, 17177 Solna, Sweden; 7 Department of Orthopaedics and Traumatology, University of Helsinki and Helsinki University Hospital, Yliopistonkatu 4, 00100 Helsinki, Finland

**Keywords:** Congenital abdominal wall defect, Cryptorchidism, Exomphalos, Gastroschisis, Omphalocele, Undescended testicles

## Abstract

**Purpose:**

Several studies have reported high prevalence of undescended testis (UDT) among boys with congenital abdominal wall defects (AWD). Due to rarity of AWDs, however, true prevalence of testicular maldescent among these boys is not known. We conducted a national register study to determine the prevalence of UDT among Finnish males with an AWD.

**Methods:**

All male infants with either gastroschisis or omphalocele born between Jan 1, 1998 and Dec 31, 2015 were identified in the Register of Congenital Malformations. The data on all performed operations were acquired from the Care Register for Health Care. The register data were examined for relevant UDT diagnosis and operation codes.

**Results:**

We identified 99 males with gastroschisis and 89 with omphalocele. UDT was diagnosed in 10 (10.1%) infants with gastroschisis and 22 (24.7%) with omphalocele. Majority of these required an operation; 8/99 (8.1%) gastroschisis and 19/89 (21.3%) omphalocele patients. UDT is more common among AWD patients than general population with the highest prevalence in omphalocele.

**Conclusions:**

Cryptorchidism is more common among boys with an AWD than general population. Furthermore, omphalocele carries significantly higher risk of UDT and need for orchidopexy than gastroschisis. Due to high prevalence testicular maldescent, careful follow-up for UDT is recommended.

## Introduction

Abdominal wall defects (AWDs), notably gastroschisis and omphalocele, are relatively rare congenital anomalies with a prevalence of 1.85 and 1.96 per 10,000 births in Finland, respectively [1, 2]. Total prevalence of these anomalies has increased in Finland since previous rates published in 1980s [3]. Due to over 50% termination rate for omphalocele, however, increased live birth prevalence has only been observed in gastroschisis [1, 2]. In most cases, gastroschisis in an isolated anomaly [1, 4, 5], whereas omphalocele is often associated with other severe anomalies including chromosomal abnormalities and cardiac defects [2, 6, 7]. As both of these congenital malformations are life-threatening conditions at birth, it has been speculated that the prevalence of UDT among boys with AWD may be under-reported because of the greater emphasis on lethal comorbidities [8].

Generally, the reported prevalence of UDT in male infants varies from 1.0 to 3.7% [9–11]. Since the introduction of Nordic consensus in 2007 [12], the mainstay of the treatment of UDT in Finland has been surgical orchidopexy before the age of one year which is supported by several studies [13, 14]. It has been postulated that the loss of intra-abdominal pressure during fetal development would predispose male infants with AWDs to testicular maldescent [15], and this theory is further supported by an animal study on newborn rats [16]. In omphalocele, co-occurrence of other major anomalies, especially brain malformations, appears to contribute to high prevalence of UDT among these patients [8, 17]. According to earlier studies, cryptorchidism appears to be more common among AWD patients [8, 18], and prevalence rates as high as 40% have been reported [19]. On the other hand, not all studies have supported these findings [20]. Furthermore, due to rarity of AWDs, only a handful of studies have explored the association of cryptorchidism and AWDs and no population-based studies are available.

Against this background, we conducted a population-based register study to determine the prevalence of UDT in infants with AWD focusing on potential differences between gastroschisis and omphalocele. Additionally, we wanted to compare our results with the reported prevalence of UDT in general population of our country. We hypothesized that patients with AWD would present with significantly higher risk of UDT than our general population.

## Methods

The data on AWDs were collected from the Finnish Register of Congenital Malformations (FRM) [21] and the Care Register for Health Care (CRHC) [22], both maintained by the Finnish Institute for Health and Welfare (THL). The FRM contains data on all live births, stillbirths, and fetuses from spontaneous abortions and terminations of pregnancy for severe fetal anomalies, all with at least one major congenital anomaly. Major structural anomalies and chromosomal defects are coded according to an extended version of the 9th Revision of the International Classification of Diseases (ICD-9) of the World Health Organization. Minor anomalies are excluded according to the system of the European Surveillance of Congenital Anomalies, EUROCAT [23]. Nationwide linkable data on all in-patient hospital discharges and outpatient visits are registered in CRHC.

FRM receives data on congenital and fetal anomalies from hospitals, health-care professionals and cytogenetic laboratories. FRM also draws data with the help of the unique personal identification code (PIC) from other national health registers: Medical Birth Register, Register on Induced Abortions, CRHC (hospital discharge data on congenital anomalies from the first two calendar years after birth), The Register of Visual Impairment, all maintained by THL, as well as from Cause-of-Death data, maintained by Statistics Finland. The data quality and coverage of these registers have been considered good in several studies [24–27].

The study population was cross-linked with the CRHC data by the PIC. Basic variables collected in CRHC include PIC (including date of birth and sex), area of residence, hospital ID, admission and discharge days, codes for operation, operation days, as well as diagnoses of patient’s medical problems. Diagnoses were recorded according to the ICD-10 and operations were registered according to the Finnish version of Nordic Medico-Statistical Committee (NOMESCO) procedure classification. All hospital admissions were analyzed between Jan 1, 1998 and Dec 31, 2015 and searched for relevant ICD-10 and operation codes for UDT.

The prevalence of UDT was compared between omphalocele and gastroschisis groups. Furthermore, using previously published Finnish cohort as a reference, we compared our results with the general prevalence of cryptorchidism in Finnish population. The reference cohort was prospectively collected in Turku University Hospital, Turku, Finland in 1997–1999 [11].

### Statistical analysis

Chi-square and Fisher’s exact tests were utilized to analyze categorical variables. A significance level of *p* < 0.05 (two-tailed) was set. Relative risk (RR) with 95% confidence interval (CI) for UDT was calculated for both AWD groups comparing results with published data in the literature. Analyses were performed using JMP Pro, version 13.1.0 for Windows (SAS Institute Inc., Cary, North Carolina, US).

### Ethical considerations

The approval of the Institutional Review board at Turku University Hospital was obtained before conducting this study. Finnish Institute for Health and Welfare gave a permission to use their health register data in this study.

## Results

We identified 189 infants with gastroschisis and 161 with omphalocele in the registers. Among these, there were 99 (52%) boys with gastroschisis and 89 (55%) with omphalocele. Mean gestational age was 36.7 (SD 1.9) weeks for gastroschisis and 37.5 (SD 3.2) for omphalocele. Infants with gastroschisis were born more prematurely than those with omphalocele (*p* = 0.041).

Ten of 99 (10.1%) boys with gastroschisis were diagnosed with UDT while 22/89 (24.7%) infants with omphalocele had evidence of testicular maldescent. In total, 29 boys underwent an operation for UDT; 8/99 (8.1%) in gastroschisis and 19/89 (21.3%) in omphalocele group. (Table 1) Bilateral operation was required in seven boys, six of these with omphalocele background. Standard groin exploration with orchidopexy was sufficient in 25 patients while two boys required a laparoscopic Fowler-Stephens procedure. Three of the five infants with UDT and no recorded operation were diagnosed during their birth admission and presumably underwent normal testicular descent. The remaining two without operation were below 6 months of age at the end of our data collection in Dec 31, 2015. Eight omphalocele infants with UDT had also other major co-morbidities including bladder exstrophy, hypospadias, renal agenesis, tetralogy of Fallot and chromosomal abnormalities, while no additional major anomalies were observed among gastroschisis patients with UDT.

**Table 1:** Prevalence of cryptorchidism in gastroschisis, omphalocele and controls without an AWD born before 37th week of gestation

	Number of patients	Undescended testicles	Surgery for undescended testicles
Gastroschisis	99	10 (10.1%)	8 (8.1%)
Omphalocele	89	22 (24.7%)	19 (21.3%)
Control group [11]	888	11 (1.2%)	N/A

Control data reported by Boisen et al. [11]

*N/A* data not available

There were significantly more UDT diagnoses and procedures required among boys with omphalocele than gastroschisis (RR 2.44, 95% CI 1.22–4.88 and RR 2.64, 95% CI 1.22–5.73, respectively). Gestational age for omphalocele infants with UDT was significantly lower than in those with normal testicles (36.0 vs. 38.0 weeks, *p* = 0.011). There was no significant difference in gestational age between those with and without UDT in gastroschisis infants (*p* = 0.76). Majority of procedures (20/27, 74%) were performed before the age of 2 years and 13/27 (48%) were operated before their first birthday. Five of those operated beyond the age of 2 years had major co-occurring anomalies.

For comparison, the prevalence of cryptorchidism in our reference cohort without an AWD was 1.0% at the age of 3 months, and among those born before 37th week of gestation there were 11/888 (1.2%) boys with UDT [11]. Boys with omphalocele and gastroschisis had significantly higher risk of UDT than general population after taking gestational age into account: RR 8.15 (95% CI 3.55–18.72) and RR 19.96 (95% CI 10.01–39.79) for gastroschisis and omphalocele, respectively.

## Discussion

In this population-based study, we have demonstrated that boys with a congenital AWD have significantly higher risk for cryptorchidism. Compared to general population, this risk is up to 8- and 20-fold in gastroschisis and omphalocele, respectively. UDT operations are often required in these infants, especially with omphalocele background.

Majority of earlier studies have reported 30–40% prevalence of cryptorchidism among boys with gastroschisis [8, 19, 28, 29]. Interestingly, this prevalence appears to be lower in Finland as reported earlier by Koivusalo et al. [18]. Their single-center series from Helsinki found 5.3% of boys with gastroschisis to have UDT and the national prevalence of UDT requiring orchidopexy in gastroschisis reported herein was 8.1%. There are geographical differences in prevalence of UDT and Finland has reported lower than average rates in earlier studies [10, 11]. We postulate that this may, at least in part, explain the lower prevalence rates in gastroschisis patients reported in our country. In gastroschisis, nonoperative initial management is recommended even with prolapsed testicles at birth, and it is reported to yield excellent results in over 50% of cases [8]. We had no data on the initial position of testicles. However, no attempt at primary orchidopexy was made in any of our cases with UDT.

Studies on the association of UDT and omphalocele are sparse. Yardley et al. [8] reported 6/27 (22%) boys with exomphalos to have UDT; bilaterally in 5 (83%) of them. Similar results were reported by Koivusalo et al. [18] where 12/75 (16%) had testicular maldescent with 5/12 (42%) bilateral conditions. Our results were consistent with published literature. We reported 21% operation rate for UDT in omphalocele group and 6/19 (31%) required bilateral orchidopexy. Contrary to the findings of the Liverpool group [8], UDT was significantly more common with omphalocele than gastroschisis in our cohort. This tendency was also supported by Koivusalo et al. [18]. Timely UDT operations were performed in majority of patients as the recommendation to operate before the age of 1 year was only placed in 2007 [12]. We postulate that co-occurring major anomalies may have caused delays in treatment in some patients. As previously reported, high prevalence of UDT among omphalocele patients appears to be caused by the condition itself as well as the associated anomalies frequently encountered [8, 17].

Prevalence of cryptorchidism in general population is higher among prematurely born infants [10, 11]. In our series, the average gestational age was around 36 weeks for both gastroschisis and omphalocele groups. As mentioned earlier, Finland also has lower than average prevalence of UDT in general population [11]. To allow reliable comparison, we selected previously published Finnish cohort [11] for comparison which reported 1.2% prevalence of cryptorchidism among male infants born before 37th week of gestation. With control group of same nationality and similar gestational age, we believe that our calculations of 8-fold risk in gastroschisis and 20-fold risk in omphalocele for UDT to be reliable.

The obvious limitation of our study was that we had no data on the outcomes of the operations for UDT. Hence, we were only able to assess the prevalence of cryptorchidism and requirement for surgery. Additionally, our control group was based on a sub-population of Finland, not the entire country, which may be a source of bias. We were also limited by reliance solely on the accuracy of the register data. On the other hand, the strength of the present study was the use of validated and high-quality register data with total population coverage [30]. Hospitals are expected to report diagnosis and operation codes accurately as they are the basis for hospital billing [22].

In conclusion, congenital abdominal wall defects are clearly associated with cryptorchidism. Also, surgery is often required both in gastroschisis and omphalocele infants with UDT. Therefore, we recommend careful pediatric surgery follow-up to arrange timely surgery when necessary.

## Data Availability

The data that support the findings of this study are available from the corresponding author upon reasonable request.
